# Supplementary material for: Access to Care and Healthcare Quality Metrics for Patients with Advanced Genitourinary Cancers in Urban versus Rural Areas
Source: Cancers (Basel). 2023 Oct 27;15(21):5171. doi: 10.3390/cancers15215171 (PMC10647451; doi:10.3390/cancers15215171)
Supplement: Supplementary file 1 [file cancers-15-05171-s001.zip › cancers-2641563-supplementary.pdf]

Supplementary Materials: Access to Care and Health Care Quality Metrics for Patients with Advanced Genitourinary Cancers in Urban Versus Rural Areas

Haoran Li [1,*], Kamal Kant Sahu [2], Shruti A Kumar [3], Nishita Tripathi [4], Nicolas Sayegh [5], Blake Nordblad [2], Beverly Chigarira [2], Sumati Gupta [2], Benjamin L. Maughan [2], Neeraj Agarwal [2], Umang Swami [2]

[1] Division of Medical Oncology, University of Kansas Cancer Center, Westwood, 66205, Kansas, United States
[2] Division of Oncology, Internal Medicine, Huntsman Cancer Institute, University of Utah, Salt Lake City, 84112, Utah, United States.
[3] Department of Internal Medicine, University of Connecticut, Farmington, 06030, Connecticut, United States
[4] Department of Internal Medicine, Wayne State University, Detroit, 48202, Michigan, United States
[5] Department of Internal Medicine, UT Southwestern Medical Center, Dallas, 75235, Texas, United States
* Correspondence: hli6@kumc.edu

Table S1. The racial composition of patients.

Cancer type	Caucasian	African American	Hispanic/ Latino	Asian	Hawaiian	Native American	Others	Total
**mPCa, n**	631	6	24	10	2	0	6	679
**mBCa, n**	177	0	6	1	0	0	0	184
**mRCC, n**	135	3	13	4	2	2	3	162
**Total, n (%)**	943 (92%)	9 (0.87%)	43 (4.19%)	15 (1.46%)	4 (0.39%)	2 (0.19%)	9 (0.87%)	1025

Table S2. Geography of urban population and distance from the cancer center.

**Distance of cancer center from patient's residence (miles)**				
	**mPCa**	**mBCa**	**mRCC**	**Total**
**0-5**	81/81	12/12	15/15	108/108
**5 to 10**	119/119	23/23	23/23	165/165
**10 to 50**	307/360	112/129	87/99	506/588
**50 to 100**	43/53	2/9	13/14	58/76
**> 100**	17/66	4/11	2/11	23/88
**Total**	567/679	153/184	140/162	860/1025

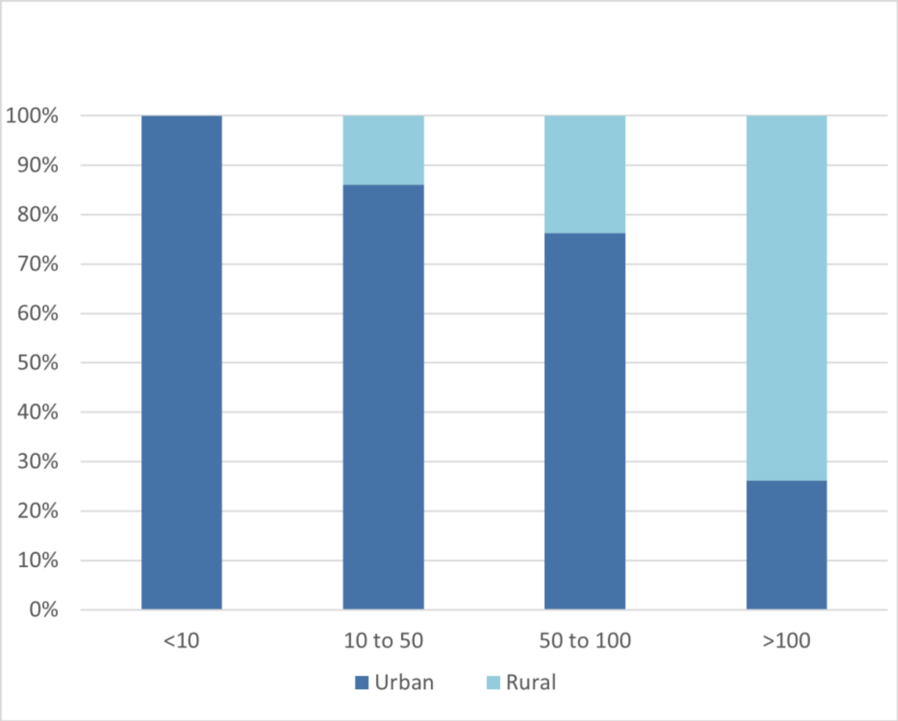

Figure S1. Distance between the patient's home and Huntsman Cancer Institute.
